# Impact of Pre-pregnancy BMI and Gestational Weight Gain on Fetomaternal Outcomes: A Cross-Sectional Analytical Study

**DOI:** 10.7759/cureus.104941

**Published:** 2026-03-09

**Authors:** Suganya Chandrasekaran, Usharani N, Arivarasan Barathi

**Affiliations:** 1 Obstetrics and Gynaecology, Thanjavur Medical College, Thanjavur, IND; 2 Community Medicine, Employees State Insurance Corporation (ESIC) Medical College and Hospital, Chennai, IND

**Keywords:** maternal morbidity, neonatal clinical outcomes, neonatal macrosomia, pre-pregnancy bmi, rate of gestational weight gain

## Abstract

Background: Pre-pregnancy body mass index (BMI) and gestational weight gain (GWG) are modifiable determinants of adverse maternal and neonatal outcomes. This study evaluated the association of BMI and GWG, based on 2009 Institute of Medicine (IOM) recommendations, with fetomaternal outcomes in a tertiary care setting.

Methods: A prospective cohort study was conducted among 300 singleton pregnant women aged 20-34 years attending a tertiary hospital. Women were categorized by pre-pregnancy BMI (underweight n=90, normal n=120, overweight n=60, obese n=30) and GWG class (appropriate n=146, excess n=56, inadequate n=98). Maternal and neonatal outcomes were compared using chi-square/Fisher’s exact tests. A p-value <0.05 was considered statistically significant.

Results: Obese women were predominantly ≥30 years (18; 60%) (p<0.001) and had higher lower-segment caesarean section (LSCS) rates (16; 53.3%) (p<0.001). Post-term delivery was common in overweight and obese groups (36; 60% and 18; 60%) (p<0.0001), while preterm delivery was higher in underweight women (35; 38.9%) (p<0.0001). Delayed wound healing increased with BMI, peaking in obese women (19; 63.3%) (p<0.0001). In GWG analysis, inadequate gain was strongly associated with preterm delivery (72; 73.5%) (p<0.001), whereas excess gain was linked to post-term delivery (36; 64.3%), induction (39; 69.6%), and LSCS (26; 46.4%) (p<0.001). Neonatally, macrosomia was highest in obese (11; 36.7%) and excess GWG (21; 37.5%) groups (p<0.001), while low birth weight was concentrated in inadequate GWG (70; 71.4%) (p<0.001). NICU admissions were highest in obese BMI (18; 60%) and inadequate GWG (68; 69.4%) (p<0.001).

Conclusion: Extremes of BMI and GWG are associated with adverse maternal and neonatal outcomes. Optimizing pre-pregnancy BMI and maintaining appropriate GWG may substantially improve fetomaternal outcomes.

## Introduction

Maternal nutritional health is a fundamental determinant of both immediate pregnancy outcomes and the future well-being of the mother and child. Among modifiable influences, pre-pregnancy body mass index (BMI) and gestational weight gain (GWG) are consistently identified as major contributors to maternal and fetal risk profiles [1]. According to the World Health Organization, a pregnancy is categorized as high-risk when identifiable factors increase the likelihood of adverse maternal or perinatal outcomes beyond those observed in the general population [2]. Pre-pregnancy obesity is one such factor, as it substantially elevates the probability of complications and therefore classifies the pregnancy as high risk.

Obesity has become a global public health crisis, with rising prevalence across both high-income and low- and middle-income countries, although patterns differ by region [3]. In the past, maternal undernutrition and low BMI were the dominant nutritional issues in pregnancy, particularly in resource-constrained settings. However, rapid urban growth, decreased physical activity, and shifts toward energy-dense diets have driven a nutritional transition. As a result, countries such as India are now experiencing increasing rates of maternal overweight and obesity alongside persistent undernutrition [4]. This shift carries serious consequences, as maternal obesity is linked to a wide range of complications, including gestational diabetes mellitus (GDM), gestational hypertension (GHT), pre-eclampsia, thromboembolic complications, higher cesarean section rates, postpartum hemorrhage (PPH), surgical site infections, and fetal macrosomia [5].

At the other end of the spectrum, women who enter pregnancy underweight (BMI <18.5 kg/m²) are also vulnerable to adverse outcomes. These include increased risks of maternal anemia, fetal growth restriction (FGR), low birth weight (LBW), and preterm birth [6]. The dual burden of risk associated with both low and high BMI highlights the need to achieve and maintain an appropriate nutritional status before conception and to monitor weight gain during pregnancy carefully. To guide clinical practice, the Institute of Medicine (IOM) developed BMI-specific recommendations for GWG intended to improve maternal and neonatal outcomes [7]. Initially issued in 1990 and revised in 2009, these guidelines specify recommended total and trimester-specific weight gain ranges for women categorized as underweight, normal weight, overweight, or obese prior to pregnancy [7,8].

The physiological basis for these recommendations lies in the complex metabolic adaptations that occur during pregnancy. Pregnancy represents a state of profound metabolic change, characterized by an early anabolic phase with increased fat stores and insulin sensitivity, followed by a late catabolic phase with progressive insulin resistance designed to shunt glucose and amino acids to the developing fetus [9]. These adaptations involve alterations in carbohydrate, lipid, and protein metabolism, mediated by placental hormones including human placental lactogen, progesterone, cortisol, and prolactin [10]. Women who begin pregnancy with abnormal BMI or who gain inappropriately during gestation may experience exaggeration of these physiological changes, leading to pathological outcomes.

India faces a unique double burden of nutritional challenges in pregnancy. While undernutrition remains prevalent, particularly in rural areas and lower socioeconomic strata, obesity is rapidly increasing in urban populations, creating a scenario where healthcare providers must address both extremes of nutritional status [4]. The National Family Health Survey data indicate that while the prevalence of underweight women of reproductive age has declined, overweight and obesity rates have nearly doubled over the past decade, signaling an urgent need for targeted nutritional interventions in antenatal care.

Despite the well-established association between BMI, GWG, and pregnancy outcomes in Western populations, there is a relative paucity of data from Indian settings. The applicability of IOM guidelines to Asian populations, who have different body composition characteristics and may experience metabolic complications at lower BMI thresholds, remains an area of active investigation. Furthermore, understanding the specific risk patterns in Indian women is essential for developing context-appropriate recommendations and interventions.

This prospective cohort study was designed to examine the relationship between pre-pregnancy BMI and GWG with maternal complications, assess their association with labour and delivery outcomes, evaluate their impact on neonatal outcomes, and quantify the risk of adverse maternal and fetal outcomes among women at the extremes of BMI and GWG. By examining these relationships in a cohort of pregnant women attending a tertiary care hospital in southern India, this study aims to generate evidence that can inform antenatal care guidelines and contribute to improved pregnancy outcomes in the Indian context. 

This article is part of a postgraduate dissertation by Dr. C. Suganya, submitted to the Tamil Nadu Dr. M.G.R. Medical University, Chennai, in partial fulfillment of the requirements for the MD (Obstetrics and Gynaecology). The work was conducted at Thanjavur Medical College, Thanjavur, Tamil Nadu, India. The thesis was published in repository in 2020.

## Materials and methods

Study design and setting

This was a prospective cohort study conducted in the Department of Obstetrics and Gynaecology at Government Health Facility in Thanjavur, Tamil Nadu, India. The study spanned a period of 12 months from January 2019 to December 2019. This is a tertiary care teaching hospital serving a predominantly rural and semi-urban population from Thanjavur district and surrounding areas. The hospital has a high patient volume and functions as a referral center for complicated obstetric cases, making it an ideal setting for studying pregnancy outcomes across different BMI categories.

The prospective cohort design was chosen as it allows for longitudinal follow-up of participants from early pregnancy through delivery, enabling accurate documentation of exposures (pre-pregnancy BMI and GWG) before the occurrence of outcomes (maternal and neonatal complications). This temporal relationship strengthens the ability to draw inferences about associations between these exposures and outcomes.

Study population and duration

The required sample size was calculated using estimates from a prior Indian study that reported the prevalence of LBW in relation to maternal BMI [11]. Assuming 80% statistical power, a 95% confidence level, and 5% absolute precision, the minimum sample size was determined to be 270 participants. A final sample size of 300 was finalized considering 10% loss to follow-up in the calculated sample size. A total of 300 eligible pregnant women meeting the predefined inclusion and exclusion criteria were recruited.

Convenient sampling method was used until the sample size was achieved. Recruitment was conducted over the first six months of the study period, with each participant followed until delivery and the immediate postpartum period. This approach ensured that all enrolled women had completed their pregnancy outcomes within the 12-month study timeframe, allowing for complete data collection and analysis.

Inclusion and exclusion criteria

Women were eligible for inclusion if they had a singleton pregnancy confirmed by ultrasound, were between 20 and 34 years of age at enrollment, attended their first antenatal visit within the first 12 weeks of gestation, and were willing to undergo regular follow-up at the study institution until delivery and the postpartum period. The age restriction was deliberately applied to reduce confounding from extremes of maternal age, which are independently associated with adverse pregnancy outcomes. Enrollment during the first trimester (before 12 weeks) was considered essential to accurately document pre-pregnancy weight and early pregnancy BMI, and to ensure standardized and consistent follow-up throughout gestation.

Women were excluded if they had a multiple pregnancy (twins or higher-order gestations), as GWG patterns and pregnancy outcomes differ substantially from singleton pregnancies. Those with pre-existing medical conditions, including overt diabetes mellitus and chronic hypertension, were also excluded because these conditions independently influence maternal and fetal outcomes. Additionally, women with severe pre-existing systemic or surgical illnesses that could affect nutritional status or the course of pregnancy were excluded. Participants with irregular follow-up or insufficient GWG data - defined as missing weight measurements at two or more scheduled antenatal visits - were not included in the final analysis. Cases with diagnosed fetal anomalies were excluded, given their potential independent effect on birth weight and neonatal outcomes. Finally, women younger than 20 years or older than 35 years were excluded in accordance with the predefined age criteria.

Ethical considerations

The research protocol received prior approval from the Institutional Ethics Committee of Thanjavur Medical College (Approval No: 558). Before recruitment, each participant was thoroughly informed about the study’s purpose, methodology, and any potential risks or benefits. Written informed consent was obtained from all participants. Strict measures were implemented to safeguard privacy, and all data were anonymized during analysis to ensure confidentiality. The study adhered to the ethical standards outlined in the Declaration of Helsinki and complied with Good Clinical Practice guidelines.

Data collection procedures

Enrollment and Baseline Assessment

All pregnant women presenting to the antenatal outpatient department for their first visit during the recruitment period were screened for eligibility. Women meeting the inclusion criteria were approached for participation, and those providing written informed consent were enrolled. At enrollment, a detailed history was obtained using a structured proforma that included demographic information (age, parity, socioeconomic status, education, occupation), obstetric history (previous pregnancies, deliveries, complications), medical history (pre-existing illnesses, medication use), and family history (diabetes, hypertension, genetic disorders). Socioeconomic status was assessed using the modified Kuppuswamy classification [12], which considers education, occupation, and income.

Anthropometric Measurements

Height was measured at the first antenatal visit using a stadiometer with the woman standing barefoot, heels together, and head positioned in the Frankfort horizontal plane. Measurements were recorded to the nearest 0.1 cm. Pre-pregnancy weight was obtained by patient recall, as is standard practice in antenatal care when women present after conception. Studies have demonstrated a reasonable correlation between recalled pre-pregnancy weight and measured weight, particularly when recall occurs in early pregnancy. For women who had documented weight measurements within three months prior to conception (from health records), this documented weight was used. Early pregnancy weight was measured at the first visit using a calibrated electronic weighing scale with the woman wearing light clothing and no footwear, recorded to the nearest 0.1 kg.

BMI Calculation and Categorization

BMI was calculated using the Quetelet index formula: BMI = Weight (kg) / Height (m²). Based on the calculated BMI, women were categorized according to the World Health Organization (WHO) classification for Asian populations, which recognizes that Asians experience metabolic complications at lower BMI thresholds. The categories were: Underweight (BMI <18.5 kg/m²), Normal weight (BMI 18.5-24.9 kg/m²), Overweight (BMI 25.0-29.9 kg/m²), and Obese (BMI ≥30.0 kg/m²) [13]. This classification allows for appropriate risk stratification in the Indian context.

Follow-up and assessment of gestational weight gain

Participants were monitored throughout pregnancy according to the routine antenatal schedule: monthly visits up to 28 weeks of gestation, fortnightly visits until 36 weeks, and weekly visits thereafter. At every visit, maternal weight was measured using the same calibrated weighing scale to ensure consistency. Gestational age was calculated from the last menstrual period and corroborated by a first-trimester ultrasound examination. Trends in weight gain were tracked longitudinally. All women received standard antenatal care, including nutritional counselling, without any additional intervention related specifically to the study.

At delivery, key obstetric details were documented, including gestational age at birth, maternal weight at admission to the labour ward, mode of delivery (spontaneous vaginal, instrumental, or cesarean), whether labour was induced and its indication, and any intrapartum complications. Total GWG was derived by subtracting the pre-pregnancy weight from the weight recorded immediately prior to delivery.

Classification of gestational weight gain

Total GWG was categorized according to the 2009 IOM recommendations, which provide BMI-specific target ranges [14]. The advised total weight gain is 12.5-18 kg for underweight women, 11.5-16 kg for those with normal BMI, 7-11.5 kg for overweight women, and 5-9 kg for obese women. Each participant’s total GWG was compared with the recommended range corresponding to her pre-pregnancy BMI category. Based on this comparison, women were grouped into three categories: inadequate GWG (below the recommended range), adequate GWG (within the recommended range), and excessive GWG (above the recommended range).

Maternal outcomes

Maternal complications evaluated included gestational diabetes mellitus (GDM), diagnosed using standard oral glucose tolerance testing between 24 and 28 weeks of gestation, and gestational hypertension (GHT), defined as new-onset blood pressure ≥140/90 mmHg after 20 weeks in the absence of proteinuria. Anaemia was defined as hemoglobin <11 g/dL during pregnancy. Amniotic fluid disorders were also recorded, with polyhydramnios defined as an amniotic fluid index (AFI) >25 cm or a single deepest pocket >8 cm, and oligohydramnios defined as AFI <5 cm or a single deepest pocket <2 cm.

Obstetric outcomes included preterm birth (<37 completed weeks), post-term delivery (>40 weeks), mode of delivery and indications for operative intervention, and PPH, defined as blood loss exceeding 500 mL after vaginal birth or 1000 mL after cesarean section. Postpartum complications such as delayed wound healing (clinically assessed during hospitalization) and thromboembolic events - including deep vein thrombosis (DVT) and cortical vein thrombosis (CVT), confirmed by imaging when clinically indicated - were also documented.

Neonatal outcomes

Neonatal data collected at birth included live birth or stillbirth status and birth weight, measured immediately after delivery using a calibrated electronic infant scale. Birth weight was categorized as LBW (<2500 g), appropriate for gestational age (AGA, 2500-3999 g), or macrosomia (≥4000 g). Apgar scores at 1 and 5 minutes were recorded. Admission to the neonatal intensive care unit (NICU) and the reasons for admission were noted. Neonatal complications assessed comprised respiratory distress syndrome (RDS), birth asphyxia, meconium-stained amniotic fluid (MSAF), and prematurity-related morbidities

Statistical analysis

Data were entered into a Microsoft Excel (Redmond, WA, USA) spreadsheet and subsequently analyzed using Stata version 17.0 (StataCorp, College Station, TX, USA). Continuous variables were expressed as mean with standard deviation (SD) for normally distributed data. Categorical variables were presented as frequencies with proportions (n, %). For comparison of categorical variables across BMI groups and GWG categories, the Chi-square test was used when expected cell frequencies were adequate. When expected frequencies were less than 5 in any cell, Fisher's exact test was employed. A p-value <0.05 was considered statistically significant.

## Results

**Figure 1:**
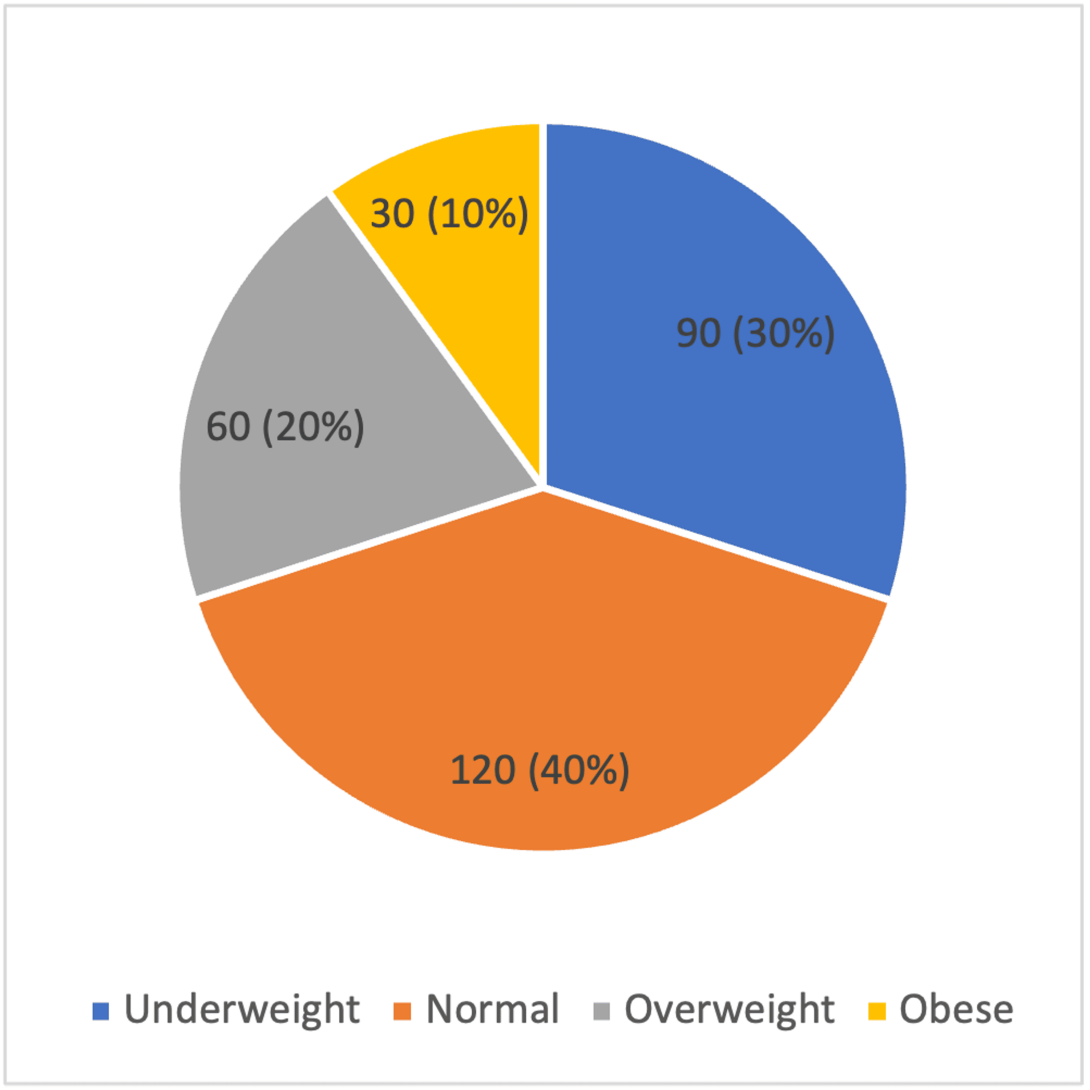
Frequency Distribution of the Type of BMI Class per WHO Classification Observed in the Study

Figure 1 shows the distribution of pre-pregnancy BMI, as per the WHO classification, among the 300 women included in the study. The largest proportion of participants had normal BMI (120; 40%), followed by underweight women (90; 30%). Overweight women constituted 60 cases (20%), while obese women formed the smallest group (30; 10%). 

**Figure 2:**
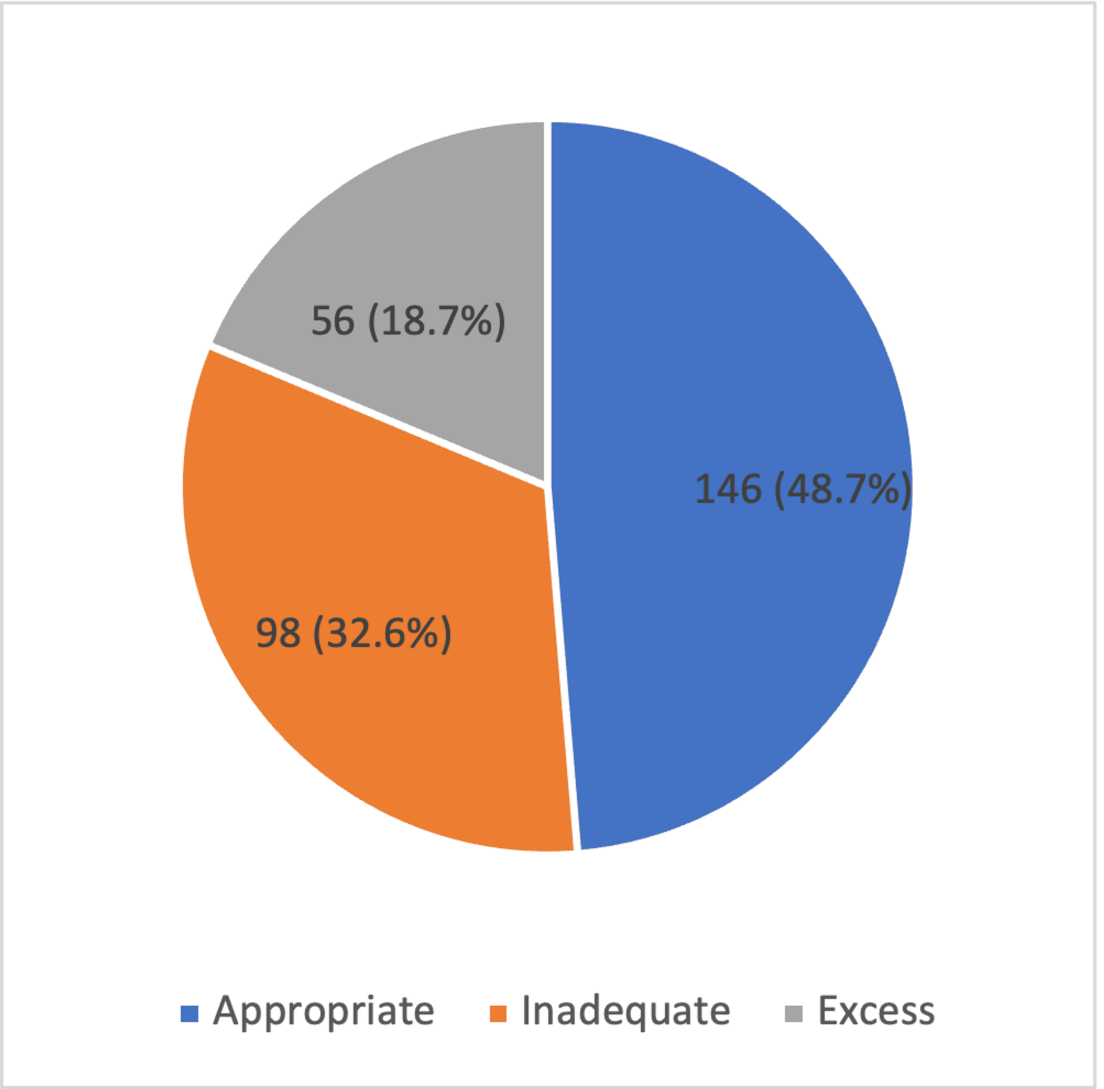
Frequency Distribution of Type of Gestational Weight Gain Based on Institute of Medicine (IOM) Criteria Observed in the Study.

Figure 2 presents the distribution of GWG according to the 2009 IOM criteria. Nearly half of the women achieved appropriate weight gain (146; 48.7%). However, 56 (18.7%) had excessive GWG, while 98 women (32.6%) had inadequate GWG. 

Table 1 presents the baseline maternal and delivery characteristics of the 300 women included in the study. Nearly half of the participants were aged 20-24 years (148; 49.3%), followed by 25-29 years (115; 38.3%), while only 37 (12.3%) were ≥30 years. Most women belonged to socioeconomic Class III (126; 42.0%), followed by Class II (92; 30.7%), whereas very few were from Class V (8; 2.6%) according to the Modified Kuppuswamy Prasad Scale 2025 [12]. The majority were primigravida (189; 63.0%), with multigravida accounting for 111 (37.0%). Regarding gestational age at delivery, 133 (44.3%) delivered at term, 102 (34.0%) had preterm delivery, and 65 (21.7%) were post-term. Vaginal delivery was the most common mode (169; 56.3%), followed by lower-segment caesarean section (LSCS) (102; 34.0%) and instrumental delivery (29; 9.7%). Induction of labour was performed in 114 women (38.0%), among whom 67 (58.8%) resulted in successful outcomes and 47 (41.2%) failed.

**Table 1:** Baseline Maternal and Delivery Characteristics of Study Participants (N = 300) *Modified Kuppuswamy Scale (2025) [12] †Outcome of induction calculated among induced cases only (N = 114). LSCS - lower segment caesarean section

Variable	Category	n (%)
Age Category	20–24 years	148 (49.3%)
	25–29 years	115 (38.3%)
	≥30 years	37 (12.3%)
Socioeconomic Status*	Class I	15 (5.0%)
	Class II	92 (30.7%)
	Class III	126 (42.0%)
	Class IV	59 (19.7%)
	Class V	8 (2.6%)
Parity	Primigravida	189 (63.0%)
	Multigravida	111 (37.0%)
Gestational Age at Delivery	Term	133 (44.3%)
	Preterm	102 (34.0%)
	Post-term	65 (21.7%)
Mode of Delivery	Vaginal (Labour natural)	169 (56.3%)
	LSCS	102 (34.0%)
	Instrumental	29 (9.7%)
Induction of Labour	Performed	114 (38.0%)
	Not done	186 (62.0%)
Outcome of Induction†	Success	67 (58.8%)
	Failure	47 (41.2%)

Table 2 shows the frequency distribution of maternal and fetal comorbidities among the 300 women studied. Anemia was the most common complication, affecting 136 women (45.3%). FGR was observed in 106 cases (35.6%), making it the most frequent fetal complication. GHT occurred in 82 women (27.3%), while oligohydramnios was noted in 77 (25.7%). GDM and PPH were each reported in 70 women (23.3%). Polyhydramnios was present in 66 cases (22.0%). Thromboembolic events (CVT/DVT) were documented in 27 women (9.0%), whereas placental abruption was the least common complication, seen in 13 cases (4.3%).

**Table 2:** Frequency Distribution of Maternal and Fetal Comorbidities (N = 300)

Comorbidity	n (%)
Anemia	136 (45.3%)
Gestational Hypertension (GHT)	82 (27.3%)
Gestational Diabetes Mellitus (GDM)	70 (23.3%)
Abruption	13 (4.3%)
Oligohydramnios	77 (25.7%)
Polyhydramnios	66 (22.0%)
Fetal Growth Restriction (FGR)	106 (35.6%)
Deep Vein Thrombosis (DVT)	27 (9.0%)
Postpartum Hemorrhage (PPH)	70 (23.3%)

Table 3 summarizes neonatal outcomes among the 300 deliveries. The majority of neonates were born alive (291; 97.0%), while stillbirths accounted for nine cases (3.0%). Regarding birth weight, 172 newborns (57.3%) were AGA, 102 (34.0%) had LBW, and 26 (8.7%) were macrosomic. Nearly half of the newborns required NICU admission (132; 44.0%), whereas 168 (56.0%) did not require intensive care support.

**Table 3:** Frequency Distribution of Neonatal Outcomes (N = 300) NICU - Neonatal Intensive Care Unit

Parameter	Category	n (%)
Outcome	Alive	291 (97.0%)
	Stillbirth	9 (3.0%)
Birth Weight	Appropriate for gestational age	172 (57.3%)
	Low birth weight	102 (34.0%)
	Macrosomia	26 (8.7%)
NICU Admission	Yes	132 (44.0%)
	No	168 (56.0%)

Table 4 demonstrates significant associations between pre-pregnancy BMI class and multiple maternal and obstetric variables. Women aged ≥30 years were predominantly obese (18; 60%) and overweight (12; 20%), whereas underweight women were largely 20-24 years old (63; 70%) (χ²=140.6, p<0.001). Higher socioeconomic status (Class II) clustered in overweight (40; 66.7%) and obese women (18; 60%), while underweight women were mainly from Class IV (39; 43.3%) (χ²=178.9, p<0.0001). Multigravidity was markedly higher among overweight (40; 66.7%) and obese women (20; 66.7%) (χ²=48.6, p<0.0001). Post-term delivery was concentrated in overweight and obese groups (36; 60% and 18; 60%, respectively), whereas preterm delivery was more frequent in underweight women (35; 38.9%) (χ²=120.3, p<0.0001). Vaginal delivery was highest among normal BMI women (92; 76.7%), while LSCS rates were highest in obese women (16; 53.3%) (χ²=102.1, p<0.001). Induction of labour was more common in overweight (35; 58.3%) and obese women (18; 60%) (χ²=37.4, p<0.001). Instrumental delivery occurred only in overweight (20; 33.3%) and obese women (9; 30%) (χ²=102.1, p<0.001). Delayed wound healing increased progressively with BMI, peaking in obese women (19; 63.3%) (χ²=63.4, p<0.0001). Overall, increasing BMI showed a clear gradient toward intervention-heavy deliveries and postoperative complications. Analysis of neonatal outcomes with respect to pre-pregnancy BMI and IOM GWG class shows clear and statistically significant associations. Among BMI groups, live birth rates were lowest in obese women (25; 83.3%), with stillbirth highest in the same group (5; 16.7%) (χ²=12.13, p<0.001). Macrosomia increased markedly with rising BMI, occurring in 14 (23.3%) overweight and 11 (36.7%) obese women, whereas it was nearly absent in underweight and normal BMI groups (χ²=84.7, p<0.001). NICU admissions were highest in obese women (18; 60%) and normal BMI women (62; 51.7%) (χ²=11.2, p=0.012).

**Table 4:** Association of Maternal and Neonatal Variables With Pre-pregnancy BMI Class (N = 300) Chi-square/Fisher's Exact Test *p-value<0.05 is statistically significant #Modified Kuppuswamy scale (2025) [12] NICU - Neonatal Intensive Care Unit; LSCS - lower segment caesarean section; LBW - low birth weight; AGA - appropriate for gestational age

Variable	Category	Underweight (n=90)	Normal (n=120)	Overweight (n=60)	Obese (n=30)	χ²	p-value
Age	20–24 yrs	63 (70%)	79 (65.8%)	6 (10%)	0 (0%)	140.6	<0.001*
	25–29 yrs	25 (27.8%)	36 (30%)	42 (70%)	12 (40%)		
	≥30 yrs	2 (2.2%)	5 (4.2%)	12 (20%)	18 (60%)		
Socioeconomic Status#	Class I	1 (1.1%)	0 (0%)	12 (20%)	2 (6.7%)	178.9	<0.0001*
	Class II	14 (15.6%)	20 (16.7%)	40 (66.7%)	18 (60%)		
	Class III	28 (31.1%)	81 (67.5%)	8 (13.3%)	9 (30%)		
	Class IV	39 (43.3%)	19 (15.8%)	0 (0%)	1 (3.3%)		
	Class V	8 (8.9%)	0 (0%)	0 (0%)	0 (0%)		
Parity	Primigravida	69 (76.7%)	90 (75%)	20 (33.3%)	10 (33.3%)	48.6	<0.0001*
	Multigravida	21 (23.3%)	30 (25%)	40 (66.7%)	20 (66.7%)		
Gestational Age at Delivery	Post-term	3 (3.3%)	8 (6.7%)	36 (60%)	18 (60%)	120.3	<0.0001*
	Preterm	35 (38.9%)	59 (49.2%)	6 (10%)	2 (6.7%)		
	Term	52 (57.8%)	53 (44.2%)	18 (30%)	10 (33.3%)		
Vaginal Delivery	Yes	55 (61.1%)	92 (76.7%)	17 (28.3%)	5 (16.7%)	102.1	<0.0001*
LSCS	Yes	35 (38.9%)	28 (23.3%)	23 (38.3%)	16 (53.3%)	102.1	<0.001*
Induction of Labour	Yes	39 (43.3%)	22 (18.3%)	35 (58.3%)	18 (60%)	37.4	<0.001*
Instrumental Delivery	Yes	0 (0%)	0 (0%)	20 (33.3%)	9 (30%)	102.1	<0.001*
Delayed Wound Healing	Yes	9 (10%)	7 (5.8%)	16 (26.7%)	19 (63.3%)	63.4	<0.0001*
Outcome	Alive	88 (97%)	118 (98.3%)	60 (100%)	25 (83.3%)	12.13	<0.001*
	Stillbirth	2 (3%)	2 (1.7%)	0 (0%)	5 (16.7%)		
Birth Weight	AGA	56 (62.2%)	60 (50%)	40 (66.7%)	16 (53.3%)	84.7	<0.001*
	LBW	(37.8%)	59 (49.2%)	6 (10%)	3 (10%)		
	Macrosomia	0 (0%)	1 (0.8%)	14 (23.3%)	11 (36.7%)		
NICU Admission	Yes	31 (34.4%)	62 (51.7%)	21 (35%)	18 (60%)	11.2	0.012*

Table 5 shows similarly strong associations between IOM GWG class and maternal-obstetric outcomes. Excess GWG was predominantly seen in women ≥30 years (30; 53.6%), while inadequate GWG was concentrated in younger women aged 20-24 years (85; 86.7%) (χ²=179.8, p<0.001). Socioeconomic Class II was common in excess GWG (28; 50%), whereas inadequate GWG clustered in Class III (58; 59.2%) and Class IV (28; 28.6%) (χ²=114.6, p<0.001). Inadequate GWG was markedly higher among primigravida (80; 81.6%) (χ²=21.6, p<0.001). Preterm delivery was strongly associated with inadequate GWG (72; 73.5%), while post-term delivery was predominantly observed in excess GWG (36; 64.3%) (χ²=157.2, p<0.001). Vaginal delivery was highest in inadequate GWG (68; 69.4%), whereas LSCS and induction of labour were most frequent in excess GWG (26; 46.4% and 39; 69.6%, respectively) (p<0.001). Instrumental delivery was also more common in excess GWG (16; 28.6%). Delayed wound healing was strikingly elevated in excess GWG (36; 64.3%) compared to appropriate (12; 8.2%) and inadequate (3; 3.1%) groups (χ²=110.2, p<0.0001). Collectively, excessive weight gain was associated with intervention-related morbidity, whereas inadequate weight gain was strongly linked to preterm delivery. Stillbirth was observed in excess (5; 9%) and inadequate GWG (4; 4%) but not in appropriate GWG (χ²=16.4, p<0.001). LBW was overwhelmingly concentrated in inadequate GWG (70; 71.4%), while macrosomia was strongly associated with excess GWG (21; 37.5%) (χ²=153.1, p<0.001). NICU admission was highest among inadequate GWG (68; 69.4%), followed by excess GWG (29; 51.8%), compared to appropriate GWG (35; 24%) (χ²=50.7, p<0.001).

**Table 5:** Association of Maternal and Obstetric Variables With Institute of Medicine (IOM) Gestational Weight Gain Class (N = 300) Chi-square/Fisher's Exact Test *p-value<0.05 is statistically significant #Modified Kuppuswamy scale (2025) [12] NICU - Neonatal Intensive Care Unit, LSCS - lower segment caesarean section; LBW - low birth weight; AGA - appropriate for gestational age

Variable	Category	Appropriate (n=146)	Excess (n=56)	Inadequate (n=98)	χ²	p-value
Age	20–24 yrs	51 (34.9%)	12 (21.4%)	85 (86.7%)	179.8	<0.001*
	25–29 yrs	90 (61.6%)	14 (25%)	11 (11.2%)		
	≥30 yrs	5 (3.4%)	30 (53.6%)	2 (2%)		
Socioeconomic Status#	Class I	3 (2.1%)	12 (21.4%)	0 (0%)	114.6	<0.001*
	Class II	60 (41.1%)	28 (50%)	4 (4.1%)		
	Class III	52 (35.6%)	16 (28.6%)	58 (59.2%)		
	Class IV	31 (21.2%)	0 (0%)	28 (28.6%)		
	Class V	0 (0%)	0 (0%)	8 (8.2%)		
Parity	Primigravida	79 (54.1%)	30 (53.6%)	80 (81.6%)	21.6	<0.001*
	Multigravida	67 (45.9%)	26 (46.4%)	18 (18.4%)		
Gestational Age at Delivery	Post-term	29 (19.9%)	36 (64.3%)	0 (0%)	157.2	<0.001*
	Preterm	26 (17.8%)	4 (7.1%)	72 (73.5%)		
	Term	91 (62.3%)	16 (28.6%)	26 (26.5%)		
Vaginal Delivery	Yes	87 (59.6%)	14 (25%)	68 (69.4%)	46.4	<0.0001*
LSCS	Yes	46 (31.5%)	26 (46.4%)	30 (30.6%)	46.4	<0.001*
Induction of Labour	Yes	55 (37.7%)	39 (69.6%)	20 (20.4%)	36.7	<0.001*
Instrumental Delivery	Yes	13 (8.9%)	16 (28.6%)	0 (0%)	46.4	<0.001*
Delayed Wound Healing	Yes	12 (8.2%)	36 (64.3%)	3 (3.1%)	110.2	<0.0001*
Outcome	Alive	146 (100%)	51 (91%)	94 (96%)	16.4	<0.001*
	Stillbirth	0 (0%)	5 (9%)	4 (4%)		
Birth Weight	AGA	114 (78.1%)	30 (53.6%)	28 (28.6%)	153.1	<0.001*
	LBW	27 (18.5%)	5 (8.9%)	70 (71.4%)		
	Macrosomia	5 (3.4%)	21 (37.5%)	0 (0%)		
NICU	Yes	35 (24%)	29 (51.8%)	68 (69.4%)	50.7	<0.001*

## Discussion

This prospective cohort study of 300 pregnant women in a tertiary care hospital in southern India provides comprehensive insights into the associations between pre-pregnancy body BMI, GWG, and fetomaternal outcomes. Our findings demonstrate significant relationships between extremes of maternal nutritional status and a wide spectrum of adverse pregnancy outcomes, reinforcing the critical importance of optimizing both pre-conception weight and GWG in the Indian population.

The distribution of BMI categories in our study revealed that women were distributed across underweight, normal, overweight, and obese categories. The combined overweight and obese group comprising a substantial proportion of the study population is particularly noteworthy, as it reflects the emerging trend of increasing obesity in urban and semi-urban Indian settings. This finding aligns with the epidemiological transition described by Nadiger and colleagues, who noted that lifestyle changes in India are rapidly increasing obesity rates, especially in urban populations [15]. The shift has significant implications for antenatal care, as healthcare providers must now address both undernutrition and overnutrition simultaneously.

Our study demonstrated a strong and progressive association between increasing BMI and GDM incidence. While a small proportion of normal BMI women developed GDM, the rates increased dramatically in overweight and obese women. The relative risk calculation revealed that obese women had a substantially increased risk of developing GDM compared to normal BMI women, while overweight women also had a markedly increased risk. These findings are consistent with the work of Doherty and colleagues, who studied 331 women and found that obesity was significantly associated with GDM [16]. The pathophysiological basis for this association lies in the progressive insulin resistance that characterizes late pregnancy, which is exaggerated in women with pre-existing increased adiposity and higher circulating levels of pro-inflammatory cytokines [17]. Women with excessive GWG also showed significantly higher GDM rates compared to those with appropriate gain, with a substantially increased relative risk.

The association between BMI and hypertensive disorders was even more pronounced in our study. GHT occurred in the majority of obese women and over half of overweight women, compared to a much smaller proportion of normal BMI women. Obese women had a markedly increased risk and overweight women a substantially increased risk of developing GHT. These findings corroborate the work of Weiss and colleagues, who reported that a majority of obese women in their cohort developed hypertensive complications [18]. The mechanism involves increased sympathetic activity, activation of the renin-angiotensin-aldosterone system, and endothelial dysfunction associated with excess adiposity [19]. Excessive GWG was also strongly associated with GHT, with the majority of excessive gainers developing hypertension compared to a smaller proportion of appropriate gainers, representing a significantly increased risk.

In contrast to the metabolic complications, anemia was predominantly associated with underweight status and inadequate GWG. Over half of the underweight women were anemic, and the majority of women with inadequate GWG had anemia. The highest anemia rates were actually observed in normal BMI women, likely reflecting the high background prevalence of nutritional anemia in this rural and semi-urban population. Adam and colleagues, studying a rural hospital in eastern Sudan, similarly found that low BMI was significantly associated with anemia and poor perinatal outcomes [20]. This finding underscores that despite the emerging obesity epidemic, undernutrition and micronutrient deficiencies remain pressing concerns in Indian antenatal populations.

A distinctive pattern emerged regarding amniotic fluid volume, with polyhydramnios predominantly affecting overweight and obese women, while oligohydramnios was more common in underweight women. Overweight women had a substantially increased risk and obese women a markedly increased risk of polyhydramnios, likely mediated through the higher rates of GDM in these groups. Conversely, underweight women had a moderately increased risk of oligohydramnios, possibly reflecting chronic dehydration and reduced placental perfusion associated with undernutrition. Excessive GWG was associated with a significantly increased risk of polyhydramnios compared to appropriate gain.

Normal vaginal delivery rates were highest among normal BMI and underweight women, declining sharply in overweight and obese women. Cesarean section rates increased progressively with BMI, with obese women having a substantially increased risk of cesarean delivery compared to normal BMI women. These findings are consistent with multiple previous studies, including those by Marie Cedergren, who demonstrated that increasing BMI is associated with higher rates of cesarean delivery independent of other risk factors [21]. The mechanisms include higher rates of labor induction, cephalopelvic disproportion due to soft tissue dystocia, and fetal macrosomia. Instrumental deliveries were also significantly more common in overweight and obese women and in those with excessive GWG compared to appropriate gainers.

PPH occurred in the majority of obese and half of overweight women, compared to a much smaller proportion of normal BMI women. Obese women had a markedly increased risk and overweight women a substantially increased risk of PPH. Delayed wound healing affected the majority of obese women (representing a markedly increased risk) and was significantly associated with excessive GWG. Thromboembolic complications (CVT/DVT) occurred exclusively in overweight and obese women, with obese women having a dramatically increased risk compared to normal BMI women. These findings align with the work of Abdollahi and colleagues, who documented that obesity significantly increases the risk of venous thrombosis through interactions with coagulation factors [22].

Birth weight showed a clear gradient across BMI categories. Macrosomia occurred in a substantial proportion of overweight and obese women, compared to a very small proportion of normal BMI women. Obese women had a dramatically increased risk and overweight women a markedly increased risk of delivering macrosomic infants. Conversely, LBW was most common in normal BMI and underweight women. These findings are consistent with the work of Ihunnaya Frederick and colleagues, who demonstrated that pre-pregnancy BMI significantly influences infant birth weight through effects on placental function and nutrient transfer [23]. Excessive GWG was associated with a markedly increased risk of macrosomia, while inadequate GWG was associated with a substantially increased risk of LBW.

NICU admission rates were highest among infants born to women with inadequate GWG and excessive GWG, compared to a smaller proportion in appropriate gainers. Stillbirths occurred exclusively in women with excessive or inadequate GWG, with no stillbirths in the appropriate GWG group. The most common indications for NICU admission were preterm birth, LBW, and respiratory distress. These findings align with a meta-analysis that underscores that inappropriate GWG, whether inadequate or excessive, compromises neonatal well-being and increases the need for specialised neonatal care [24].

The study had certain inherent limitations. First, pre-pregnancy weight was primarily based on maternal recall, which may introduce recall bias, although early pregnancy recall has been shown to correlate reasonably well with measured pre-pregnancy weight. Second, the study was conducted at a single tertiary care centre, which may limit the generalizability of the findings to other populations. Third, although the prospective cohort design allowed temporal assessment of exposures and outcomes, the analysis relied mainly on univariate comparisons. Multivariable regression analysis to adjust for potential confounders such as maternal age, parity, and comorbidities was not performed because several outcome events were unevenly distributed across BMI and gestational weight gain subgroups, which could result in unstable estimates and model overfitting. Finally, residual confounding from unmeasured factors cannot be entirely excluded. Larger multicenter studies with adequate event distribution are needed to allow robust multivariable modelling and confirm these findings.

The findings of this study have several important implications for clinical practice in India. First, routine calculation of pre-pregnancy BMI should be mandatory at the first antenatal visit, and women should be counseled regarding appropriate weight gain based on IOM guidelines. Second, the high rates of GDM and GHT in overweight and obese women support the need for enhanced surveillance, including early and repeated screening for these conditions. Third, the strong association between inadequate GWG and adverse outcomes (anemia, LBW, preterm birth) in underweight women indicates the need for targeted nutritional interventions and close monitoring of weight gain in this group. Fourth, the high rates of cesarean delivery and postpartum complications in obese women suggest that delivery planning should include multidisciplinary involvement and preparation for potential complications. Fifth, the neonatal outcomes highlight the importance of appropriate GWG in reducing NICU admissions and improving infant health.

At a broader level, these findings support the integration of pre-conception counselling and nutritional optimization into maternal health programs. The dual burden of undernutrition and overnutrition in India requires nuanced approaches that address both extremes. For underweight women, interventions should focus on nutritional supplementation and achieving adequate weight gain before and during pregnancy. For overweight and obese women, pre-conception weight loss programs and close monitoring of GWG are essential. The IOM guidelines provide a useful framework, but their applicability to Indian populations should be further validated through larger multicenter studies.

## Conclusions

This prospective observational study demonstrates that extremes of pre-pregnancy BMI and inappropriate GWG are significantly associated with a wide range of adverse maternal and neonatal outcomes in the Indian population. Overweight and obese women, as well as those with excessive GWG, face substantially increased risks of GDM, GHT, polyhydramnios, cesarean delivery, PPH, delayed wound healing, thromboembolic events, and macrosomia. Conversely, underweight women and those with inadequate GWG are at higher risk of anemia, oligohydramnios, fetal growth restriction, low birth weight infants, and increased NICU admissions. These associations persisted across multiple outcomes with large effect sizes, underscoring the clinical significance of maternal nutritional status. The findings highlight the critical importance of routine BMI assessment, individualized counseling on appropriate GWG based on IOM guidelines, and targeted surveillance for complications in high-risk groups. As India navigates the dual burden of undernutrition and emerging obesity epidemics, integrating nutritional optimization into routine antenatal care is essential for improving pregnancy outcomes and achieving maternal and child health goals.
